# Unveiling the Group A Streptococcus Vaccine-Based L-Rhamnose from Backbone of Group A Carbohydrate: Current Insight Against Acute Rheumatic Fever to Reduce the Global Burden of Rheumatic Heart Disease

**DOI:** 10.12688/f1000research.144903.2

**Published:** 2024-10-28

**Authors:** Ade Meidian Ambari, Faqrizal Ria Qhabibi, Dwita Rian Desandri, Bambang Dwiputra, Pirel Aulia Baravia, Indira Kalyana Makes, Basuni Radi

**Affiliations:** 1 Cardiology and Vascular Department, Faculty of Medicine, University of Indonesia, Jakarta, Jakarta, 10430, Indonesia; 2 Cardiovascular Prevention and Rehabilitation Department, National Cardiovascular Center Hospital Harapan Kita, Jakarta, Jakarta, 11420, Indonesia; 3 Research Assistant, National Cardiovascular Center Hospital Harapan Kita, Jakarta, Jakarta, 11420, Indonesia; 4 Cardiovascular Prevention and Rehabilitation Department, Dr. Saiful Anwar General Hospital, Malang, East Java, 65122, Indonesia

**Keywords:** Acute rheumatic fever; Rheumatic heart disease; Group A Streptococcus; L-rhamnose; Vaccine

## Abstract

Group A Streptococcus (GAS) is a widely distributed bacterium that is Gram-positive and serves as the primary cause of acute rheumatic fever (ARF) episodes. Rheumatic heart disease (RHD) is a sequela resulting from repeated ARF attacks which are also caused by repeated GAS infections. ARF/RHD morbidity and mortality rates are incredibly high in low- and middle-income countries. This is closely related to poor levels of sanitation which causes the enhanced incidence of GAS infections. Management of carditis in RHD cases is quite challenging, particularly in developing countries, considering that medical treatment is only palliative, while definitive treatment often requires more invasive procedures with high costs. Preventive action through vaccination against GAS infection is one of the most effective steps as a solution in reducing RHD morbidity and mortality due to curative treatments are expensive. Various developments of M-protein-based GAS vaccines have been carried out over the last few decades and have recently begun to enter the clinical stage. Nevertheless, this vaccination generates cross-reactive antibodies that might trigger ARF assaults as a result of the resemblance between the M-protein structure and proteins found in many human tissues. Consequently, the development of a vaccine utilizing _L_-Rhamnose derived from the poly-rhamnose backbone of Group A Carbohydrate (GAC) commenced. The _L_-Rhamnose-based vaccine was chosen due to the absence of the Rhamnose biosynthesis pathway in mammalian cells including humans thus this molecule is not found in any body tissue. Recent pre-clinical studies reveal that _L_-Rhamnose-based vaccines provide a protective effect by increasing IgG antibody titers without causing cross-reactive antibodies in test animal tissue. These findings demonstrate that the _L_-Rhamnose-based vaccine possesses strong immunogenicity, which effectively protects against GAS infection while maintaining a significantly higher degree of safety.

## Introduction

**Figure 1.**
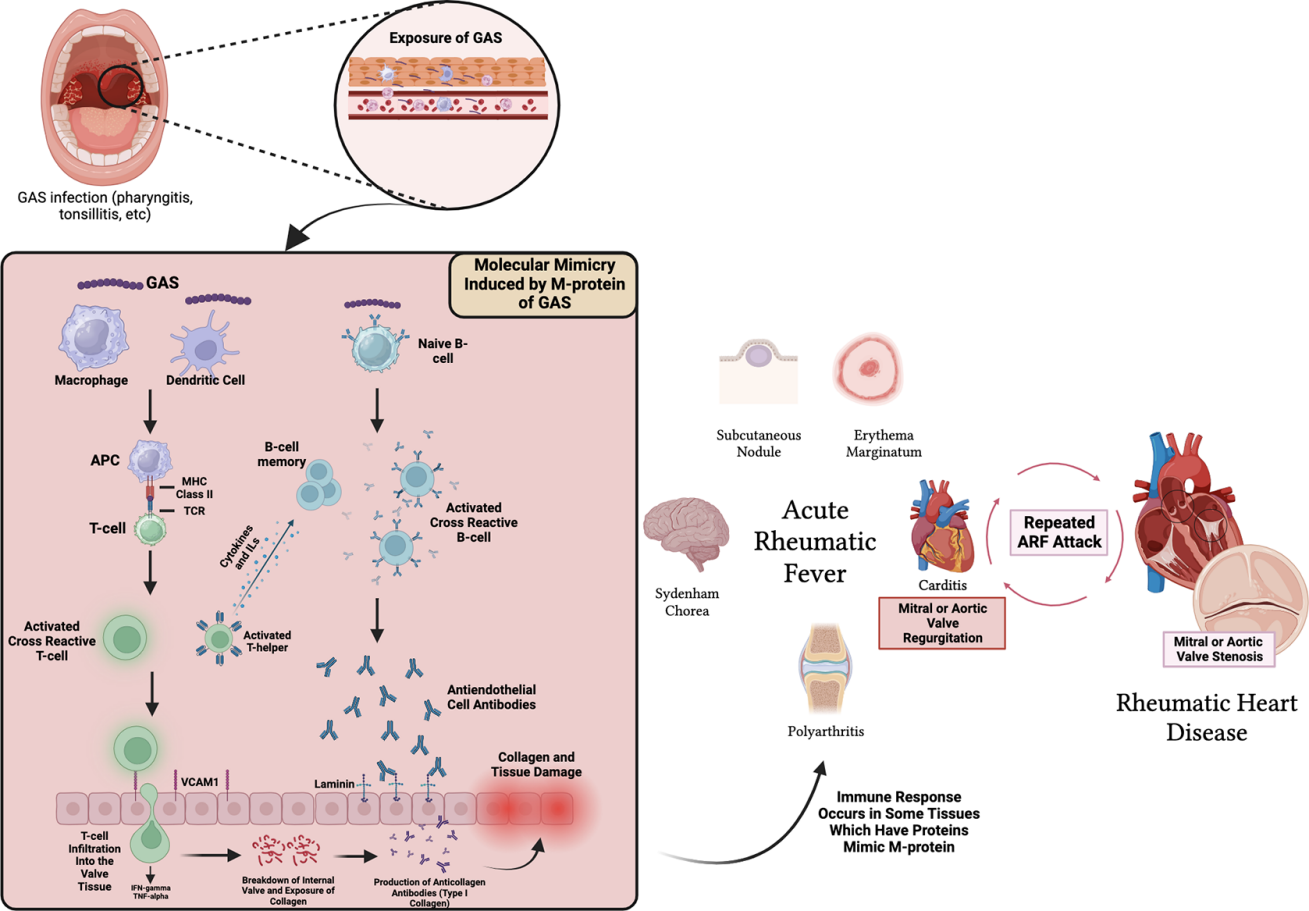
Pathogenesis of ARF in heart and related tissues. MHC = Major Histocompatibility Complex, TCR = T-cell Receptor, IL = Interleukin, VCAM-1 = Vascular Cell Adhesion Molecule-1.

Group A Streptococcus (GAS) is a cosmopolitan bacterium that has become the etiology of various human diseases, from mild illnesses such as tonsilitis and impetigo, to severe ones such as scarlet fever, toxic shock, and necrotizing fasciitis. Acute rheumatic fever (ARF) is an inflammatory reaction triggered by Group A Streptococcus infection which usually occurs approximately two to three weeks following a sore throat illness. ^1^^,^^2^ ARF is characterized by several clinical symptoms consisting of polyarthritis migrant (35-88%) and carditis (50-78%) which usually cause mitral or aortic valve regurgitation. Besides that, other signs that usually accompany ARF include abnormal involuntary movement or Sydenham chorea (2-19%), erythema marginatum (<6%), subcutaneous nodule (1-13%), and increased laboratory values such as erythrocyte sedimentation rate, neutrophils, and CRP. ^2^^–^^5^ These clinical symptoms are used in establishing the diagnosis of ARF which is concluded in Jones criteria which was first proposed in 1944 and has undergone several revisions until the American Heart Association (AHA) issued the latest revision in 2015 which divides these clinical syndromes into major and minor criteria and dividing the population based on where they live whether they are classified into low, medium, or high-risk areas. ^3^^,^^6^ Various clinical manifestations that appear during ARF are the result of autoantibodies against tissues found in the joints, brain, and heart due to the similarity of the molecular structure of protein found in body tissues with the molecular structure of M-protein antigen found in GAS. ^7^^,^^8^ The structural similarity between α-helical coiled M-protein antigens in GAS and various body proteins that cause autoantibodies is referred to as molecular mimicry ( Figure 1). Molecular mimicry between Streptococcus and the heart reveals a cross-reactive antibody that recognizes several types of epitopes found on Streptococcus M-protein and protein found on heart valves. ^8^^,^^9^ In the latest development, the concept of “neo-antigen” theory emerged and explains that GAS gained access to the sub-endothelial collagen matrix, where the peptide associated with rheumatic fever (PARF) M-protein domain binds to type IV in the CB3 region thereby creating a neo-antigen that can induce an autoimmune response against collagen. ^10^^,^^11^ Both molecular mimicry and neo-antigen theories are still being further researched, however, repeated ARF will cause tissue scarring as a result of response to pro-inflammatory cytokines such as interleukin-1 (IL-1), IL-12, and tumor necrosis factor-α (TNF-α) which increased when ARF occurs and ultimately causing tissue fibrosis. ^12^^,^^13^ Fibrosis in cardiac valves, notably the mitral valve, leads to valve regurgitation, which progresses to stenosis. Mitral regurgitation (MR) to mitral stenosis (MS) conversion is a pathognomonic sign of rheumatic heart disease (RHD). ^12^^,^^14^^,^^15^

Rheumatic heart disease (RHD) is a myocardial pan-carditis and the cardiac sequela of ARF. Although ARF is self-limiting, inflammation in heart valves caused by a single or repeated episode of ARF can lead to scarring and chronic valve dysfunction. ^16^^,^^17^ Chronic rheumatic heart disease (RHD) is clinically identified by the occurrence of fibrinous pericarditis and interstitial granulomas or Aschoff’s nodules. These nodules consist of clusters of loose granulomas with necrosis of central fibrinoids, macrophages, B cells, and multinucleated giant cells in the myocardium. The myocardial damage caused by RHD can improve without leaving any lasting harm, nevertheless, if accompanied by valvulitis, it leads to permanent damage. ^18^^,^^19^ The diagnosis of carditis due to RHD based on the finding of diastolic murmur sound through auscultation at the apex of the heart is categorized as clinical carditis. Meanwhile, the establishment of carditis through echocardiography examination due to normal findings during physical examination is referred to as subclinical carditis. ^20^^,^^21^ Heart valve-related RHD is the most serious clinical manifestation and has been the focus of numerous studies for the last several decades. ^9^^,^^22^^–^^24^ In symptomatic severe valvular cases, ideally, treatment for RHD requires surgery or catheter-based treatment. Pharmacological therapy is typically employed solely to address heart failure or atrial fibrillation (AF), which often arises from valve regurgitation or stenosis related to rheumatic heart disease (RHD). ^21^ For patients with severe symptomatic mitral stenosis, percutaneous mitral balloon commissurotomy (PBMC) is the main treatment of choice. ^21^^,^^25^^,^^26^ PBMC treatment is a main option, particularly in low and middle-income countries, due to its lower cost compared to valve surgery and a success rate above 95%. ^27^ Nonetheless, there are several contraindications including left atrial clots or extensive calcium deposits, that render PBMC inadvisable; thus, the sole alternative is valve surgery, which is considerably more costly. ^21^^,^^26^ The high cost of curative treatment for RHD arises from the absence of pharmacological treatment to address valve problems, rendering costly invasive procedures the sole alternative. Therefore, preventive measures have a vital role in efforts to reduce the global burden of RHD. RHD prevention and control is broadly divided into three parts, including primordial prevention, primary prevention, and secondary prevention. ^21^^,^^28^^,^^29^ Vaccination serves as a preventive measure against ARF and RHD by providing targeted protection against GAS infection. ^30^ Therefore, vaccine development is very promising as a big step to diminish the incidence of ARF and RHD, particularly in low- and middle-income countries which have worse sanitation and socioeconomic levels than high-income countries, which ultimately affects the rate of GAS infection.

## Discussion: Current Global Epidemiology of GAS, ARF, and RHD

GAS ranks among the top ten causes of infection-related death worldwide. GAS infections predominantly occur in low and middle-income (developing) nations, where factors such as overcrowding, inadequate nutrition, unsatisfactory sanitation, and substandard living conditions are believed to contribute to the widespread occurrence of GAS disease. ^31^^,^^32^ GAS is responsible for approximately 700 million cases of pharyngitis per year globally. An estimated minimum of 517,000 fatalities are attributed to severe GAS infections each year, with a minimum prevalence of 18.1 million cases and an annual increment of 1.78 million new cases. Meanwhile, the burden of invasive GAS (iGAS) cases is very high, with 600,000-663,000 new cases reported every year with a mortality rate of 25% or around 163,000 deaths per year. ^32^^–^^35^ Streptococcal pharyngitis is a very common infection in children. Streptococcus in susceptible hosts can trigger an abnormal inflammatory immune response due to the cross-reactivity of Streptococcal antibodies to myocardial, synovial, and basal ganglia tissue, thus becoming the main etiology of ARF. ARF which is a sequela of GAS infection primarily manifests in children and adolescents. The peak incidence of ARF is between 5 to 15 years, and it is exceedingly rare around 30 years of age. Epidemiologically, ARF occurs in all parts of the world. ^8^^,^^36^ In every single year, approximately 500,000 new cases of ARF are reported worldwide. ^2^^,^^37^ A study conducted by the World Health Organization (WHO) reveals that the total global burden of acute rheumatic fever (ARF) cases amounts to 471,000 cases annually. Among children aged 5 to 15 years old, the incidence of ARF is 10 cases per 100,000 in industrialized countries and 374 cases per 100,000 in Pacific countries. ^32^ From an economic perspective, the financial burden of ARF is substantial; for instance, in the USA, it is estimated that the annual cost of ARF ranges from 224 to 539 million USD. ^4^

Around 60% of individuals residing in endemic populations who have ARF will ultimately develop RHD. RHD is a serious consequence of inappropriate GAS infection management resulting in recurrent ARF attacks. According to the Institute for Health Metrics and Evaluation (IHME) Global Burden of Disease report, the global prevalence of RHD exceeds 40 million cases, primarily concentrated in low- and middle-income nations. ^38^ RHD has a substantial impact on the morbidity and mortality rates in low- and middle-income countries, resulting in over 300,000 deaths annually and more than 10 million disability-adjusted life years. ^39^ According to data released in 2013, the prevalence of RHD cases is 33 million, with an annual death rate of 275,000, dominated by low- and middle-income countries. The prevalence of RHD escalates with age, and the survival rate is determined upon access to and adherence to secondary prophylaxis to prevent ARF recurrence, the severity of valve damage, and the availability of specialists and surgical intervention. ^32^^,^^39^^,^^40^ The epidemiology of RHD varies in each region, the prevalence is quite high in the Pacific ^41^ and Africa, ^42^^,^^43^ but high burden in Latin American country, ^44^ the Middle East, ^45^ and Asia. ^32^ Meanwhile, in industrialized countries such as the USA, the incidence of RHD is exceedingly low (0.04 cases per 1000 children). ^46^ An estimate shows that 40% of people with RHD remain oblivious to an ARF episode, meaning they are unaware of having experienced an ARF attack. Consequently, RHD is only detected when individuals show cardiac symptoms that appear late in the disease. ^15^^,^^47^ If we draw a common thread from the high prevalence, morbidity, and mortality rates of RHD, it cannot be separated from the presence of GAS infection as the main etiology of ARF. Worldwide, Group A Streptococcus (GAS) continues to exhibit sensitivity to penicillin, despite evidence indicating that penicillin has not been successful in completely eliminating GAS-related pharyngitis and tonsillitis. In addition, GAS remains susceptible to other beta-lactam antibiotics such as amoxicillin and cephalosporins. ^48^^,^^49^ Recent research has shown an uncommon mutation in penicillin-binding protein (PBP) 2B in two strains of GAS, which decreases their vulnerability to beta-lactam antibiotics. ^50^ This serves as a warning that GAS is progressing towards resistance against commonly administered antibiotics, such as penicillin and amoxicillin. Consequently, the most promising strategy for combating antibiotic resistance in GAS, the main etiology of ARF that can develop into RHD, development and implementation of a GAS vaccine.

## GAS Vaccine Development for ARF/RHD

A vaccine for GAS would theoretically be the most cost-effective intervention in an ARF/RHD endemic country. An economic assessment carried out in the USA showed that the implementation of the vaccination program would reduce the GAS infection rate by up to 20% in all age groups, thereby saving costs of approximately 1 billion USD. ^51^ Furthermore, the development of an effective GAS vaccine will provide several potential benefits including protection from infection and diminishing usage of antibiotics thereby reducing the rate of microbial resistance. ^52^^,^^53^ However, nowadays there is no licensed GAS vaccine, and it remains in the developmental phase. In 2018, WHO issued a roadmap from the development of the first GAS vaccine. ^54^ The primary obstacle in developing a successful and secure GAS vaccine has been constant in recent decades, which is the production of universally applicable vaccine candidates capable of safeguarding against both existing and future strains of GAS. ^55^ The result of GAS genome sequencing shows two major issues in vaccine development, namely (i) extensive genome heterogenicity of gas isolates which is the result of frequent genetic recombination events such as gene exchange and single nucleotide polymorphism (SNP), and (ii) variations in protein sequences. ^56^^,^^57^ A recent extensive genomic study showed that there are 13 candidate antigenic proteins that are conserved in more than 99% of GAS isolates found globally. ^1^^,^^58^ Several candidates are still in the safety and immunogenicity testing stage in rodents or experimental mice and rabbits, while others are beginning to enter clinical trial development on human subjects. Antigenic protein candidates are broadly classified into two groups: (i) M-protein-based candidates and (ii) non-M-protein candidates. ^1^^,^^59^^–^^61^

M-protein is an immunodominant GAS protein encoded by the emm gene. The M-protein structure comprises a coiled-coil structure that extends 600 nm and is anchored in the bacterial cell wall. ^59^^,^^60^^,^^62^^,^^63^ M-protein has been widely studied and is believed to have a role in adhering to host cells and blocking phagocytosis thereby helping GAS colonization. ^63^^,^^64^ M-protein is a versatile protein in terms of both structure and function. M-protein can bind to fibrinogen, fibronectin, and host plasminogen, apart from that it also plays a role in interfering with complement protein deposits through binding to the Fc domain with IgG and complement regulators namely C4BP protein and factor H. Consequently, M-protein significantly contributes to virulence factors by resisting opsonophagocytosis. ^65^^–^^67^ Recent investigations have shown a potential GAS vaccine candidate resulting from the development of vaccines containing the N-terminal or C-terminal domains of the M protein, or a combination thereof, demonstrating protective effectiveness against GAS infections. ^1^ To date, there have been three GAS vaccine candidates recorded on globaldata.com that have been tested in the clinical trials phase or are scheduled for clinical trial phase I. ^1^^,^^33^ In 2020, a phase I clinical trial was carried out using *StreptAnova* targeting M-protein. *StreptAnova* is a 30-valent vaccine candidate designed using the N-terminal peptide from 30 M-protein. ^1^^,^^68^^,^^69^ Individuals who received StrepAnova developed functional (opsonophagocytic) antibodies, although protection from this vaccine has not been conclusively proven in animal challenge models. ^70^ Meanwhile, other M-protein based vaccines such as *StrepInCor* contain a peptide consisting of 55 amino acids from the C-peptide region of the M protein. This region is the most conserved part among the GAS serotypes. ^71^ *StrepInCor* vaccination in phase I clinical trials will begin in late 2023. ^33^ One of the most promising clinical trial approaches currently is the use of a combination of two synthetic peptides, namely two M-protein epitopes (modified p*17 and J8) which have been paired with an epitope of the streptococcal anti-neutrophil factor, Spy-CEP (K4S2). ^1^^,^^33^ Test results on pre-clinical models showed no cardiac or neurological pathologies so it was initiated into clinical trials in 2022. However, the primary challenge of developing an M-protein-based GAS vaccine is the potential for cross-reactivity with human tissues, particularly the myosin protein found in cardiac muscle cells. ^64^^,^^72^^,^^73^ The first evidence showing the existence of cross-reactivity between anti-streptococcal antibodies and human heart tissue was found in experimental mice that had been immunized with GAS components. In fact, cross-reactivity has become one of the main hypothesized mechanisms causing the emergence of ARF attacks several weeks after the onset of manifestations of GAS infection such as sore throat infection or impetigo due to autoimmunity which recognizes the M-protein GAS along with the proteins found in the heart valves, synovial tissue, and basal ganglia due to the similarity of protein structures which is referred to as molecular mimicry. ^8^^,^^74^^–^^77^

The growing interest in developing GAS vaccines based on non-M protein antigens stems from a desire to avoid GAS vaccine targets that may cause autoimmunity. The development of non-M protein vaccines began in the 1990s using C5a peptidase (Streptococcal C5a protease, SCPA). ^78^ Apart from SCPA, other non-M protein GAS vaccine candidates include Streptolysin O (SLO), Streptococcal Pyrogenic Exotoxins, and multi-component vaccines. ^33^ All of these vaccine candidates are still in the pre-clinical trial testing phase. The main challenge in developing non-M protein-based vaccines is low coverage against all GAS strains globally. In addition, to achieve optimal protection against all GAS isolates, the various protein components in the vaccine must be present in all GAS isolates or strains.

## Development of GAC Vaccine based on Poly-rhamnose Backbone from Group A Carbohydrate (GAC)

### Structure and Biosynthesis of *Group A Carbohydrate* (GAC)

**Figure 2.**
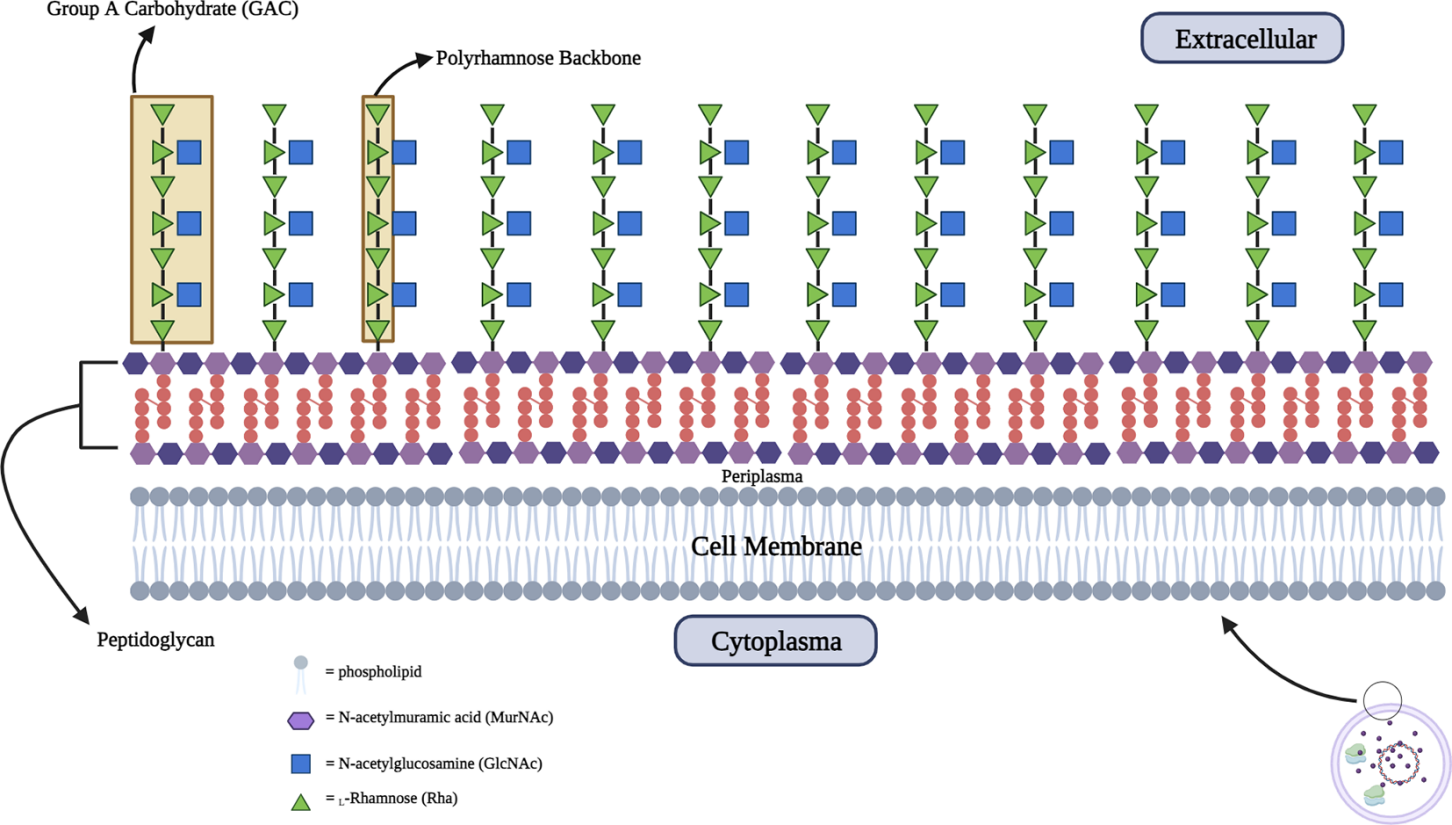
Schematic structure of Group A Carbohydrate (GAC) which embedded into peptidoglycan with extracellular presentation.

The surface polysaccharide known as Group A Carbohydrate (GAC) is present in all identified serotypes and is completely conserved. It is composed of repeating trisaccharide units with the main chain formed by *α*-1,2- and *α*-1,3-linked l-rhamnose (Rha) residue and a *β*-1,3-linked N-acetyl- d-glucosamine (GlcNAc) residue as a side chain embedded into the 3-O-position of the latter Rhamnose residue. ^56^^,^^79^^–^^81^ The GAC component is abundant and essential in GAS cells because it forms approximately 40-60% of the mass of the cell wall which has a function in structural support as a barrier against the environment and maintaining cell morphology. ^79^ GAC forms covalent bonds with N-acetylmuramic acid (MurNAc) which is the main component of peptidoglycan. As previously mentioned, the GAC component is 100% conserved because all GAS serotypes express GAC which consists of a poly-rhamnose backbone with an N-acetylglucosamine (GlcNAc) side chain with repeating trisaccharide units [3) α- l-Rhap (1➔2) [β- d-GlcpNAc(1➔3) α- l-Rhap(1➔3)] _n_. The backbone of GAC consists of l *-rhamnose* repeat units linked by *α-1,3-α-1,2 glycosidic* ( Figure 2). ^82^^–^^85^ The reported average molecular mass of GAC is 8.9 ± 1 kDa, attributed to the 18-unit repeat. ^56^

A molecular study shows the biosynthetic mechanism of GAC where the rhamnan (Rha) polymer is arranged on the cytoplasmic side of the plasma membrane and then translocated to the cell surface by the ABC transporter protein and modified by GlcNAc on the outside of the cell membrane. A lipid carrier, namely GlcNAc-P-P-Und, acts as an acceptor for the initiation of rhamnan backbone biosynthesis. ^86^^,^^87^ Recent studies show that there are 12 gene clusters that play a role in expressing various enzymes and functional proteins that play a role in the GAC biosynthesis process. A series of steps in the rhamnan biosynthesis process involve the action of glycotransferases encoded by the GacB, GacC, GacG, and GacF genes so that the rhamnan polymer that forms the backbone will become a structure commonly known as poly-rhamnose. ^87^^–^^89^ GacA is the first gene of the GAC gene cluster, but the GacA gene encodes an essential enzyme that has a function in catalysing the last step of the four steps of the dTDP- l-Rhamnose biosynthesis pathway during the production of poly-rhamnose GAC cores. Meanwhile, the GacD and GacE genes encode transporter proteins that play a role in translocation of rhamnan polymer from the cytoplasmic side of the membrane to the outer surface of the cell membrane. ^80^^,^^88^ The biosynthesis of the rhamnan backbone, a component of GAC, is essential for the viability of GAS. Anchoring of GlcNAc to the poly-rhamnose chain to form the GAC structure involves a protein encoded by the GacL gene. GlcNAc is tethered to the 2-hydroxyl of the rhamnose residue so that GlcNAc presentation leads out of the rhamnose helix. ^56^ Approximately 20 – 30% of the GlcNAc residues in GAC polymers contain modified components from glycerol phosphate. ^56^^,^^84^ These modifications are mainly found in the C6-hydroxyl group. The location of the GAC polymer is located on the outer surface of the GAS cell wall and is highly conserved among GAS strains which makes GAC interesting polysaccharide component of glycoconjugate vaccines. ^79^^,^^89^

### GAC Role Towards GAS Virulence

A known mutant group A streptococcus without the presence of GlcNAc has susceptibility to leukocytes, including neutrophils that show much greater binding ability to bacteria. This is caused by cathelicidin, which is a human peptide defence, which has a higher affinity for mutant polysaccharides. ^83^^,^^90^ Furthermore, the absence of GlcNAc has a major influence because GlcNAc has a function in reducing the binding of cationic bactericidal enzyme human Group IIA. ^84^^,^^88^^,^^91^ Therefore, the GlcNAc side chain is the most important virulence determinant in GAS. The polysaccharide branch consisting of rhamnose and GlcNAc side chains has shown immunogenic capabilities that have been demonstrated through rabbit and human antisera, nuclear magnetic resonance (NMR) techniques demonstrating the interaction of GAC with mAb and computer simulations. ^82^ The GlcNAc side chain is predicted to have a key role in the evasion of the human immune system. The GlcNAc side chain of GAC contributes to resistance to innate immunity and the virulence phenotype of globally distributed GAS strains. In GAS serotype M1, the absence of GlcNAc results in significantly reduced survival in the blood of humans and animals modelled for infection. ^92^ Meanwhile, in GAS serotype M3, the GlcNAc side chain is required to enhancing survival in human blood through platelet release. ^83^ The characteristics of resistance to neutrophil killing in GAS are more conserved in GAS strains M1, M2, M3, and M4 with the GlcNAc side chain of GAC. ^48^^,^^83^^,^^92^

### Immunogenicity of GAC

A study conducted by Sabharwal *et al.* demonstrated that GAC is immunogenic and protective against GAS infection. In addition, antibodies against the presence of GAC in human serum are highly opsonic against various M-protein serotypes of GAS and are not cross-reactive against human tissues. ^93^^,^^94^ Another study conducted by Pinto *et al.* also showed that the size and orientation of the antigenic epitope of GAC are crucial parameters for recognition by both monoclonal and polyclonal antibodies so that antibodies induced by GAC-based vaccines will effectively recognize epitopes on the surface of GAS cell walls. ^95^ Both Pinto and Kabanova *et al.* simultaneously demonstrated that the repeat unit of GAC is an optimum epitope when used as a vaccine component to elicit an adequate immune response against GAS infection. ^95^^,^^96^ Meanwhile, Wang and colleagues showed that three repeat units in the GAC structure conjugated to the inactive form of group A streptococcal C5a peptidase (ScpA193) showed efficient and feasible results if developed as a vaccine against GAS infection. ^94^ However, several studies in pre-clinical studies show evidence that antibodies formed from pure GlcNAc-based GAC vaccines have autoimmunity capabilities due to cross-reaction with cardiac myosin protein. ^97^^–^^100^ The study conducted by Galvin *et al.* found that monoclonal antibodies derived from patients with rheumatic carditis and arthritis were cross-reacted to GlcNAc from GAC residues. ^101^^,^^102^ Antibodies that recognize GlcNAc from GAC residues are the main cause of major clinical neuronal disorders from acute rheumatic fever such as Sydenham chorea. This is confirmed by an in-vivo study conducted by Brimberg *et al.* used a mouse model that demonstrated the presence of streptococcal antibodies deposited in the frontal cortex, striatum, and thalamus. ^103^ Therefore, the utilization of the GlcNAc component from GAC residues in vaccine development will face the same challenges as developing an M-protein-based GAS vaccine, namely the presence of cross-reactivity that can arise against human body tissue.

### Rhamnose-based GAS Vaccine from GAC Backbone

l-Rhamnose (Rha) is a deoxy sugar due to the hydroxyl group on one of the carbon atoms of the sugar carbon chain is replaced with a hydrogen atom. ^81^^,^^104^ Rhamnose is very common in bacteria and plants. Rhamnose is an essential monomer for pathogenic bacteria because it is the main component of cell walls and capsules which has a vital role in virulence and bacterial survival. ^83^^,^^105^ l-Rhamnose is synthesized by bacteria from glucose-1-phosphate (Glu-1-P) which acts as a precursor through the enzyme glucose-1-phosphate thymidylyltransferase (RmlA) which catalyses the transfer process of thymidyl monophosphate nucleotide to Glu-1-P. ^86^ Afterward, the enzyme dTDP- d-glucose 4,6-dehydratase (RmlB) catalyses the deoxidation process of the hydroxyl group on C4 of the sugar ring followed by dehydration (release of H _2_O). The third enzyme, namely dTDP-6-deoxy- d-xylo-4-hexulose 3,5-epimerase (RmlC), catalyses the double epimerization reaction at the C3 and C3 positions of the sugar ring. Next, the final step, namely the enzyme dTDP-6-deoxy- d-xylo-4-hexulose reductase (RmlD) reduces the keto function on C4 to form the final product, namely dTDP- l-Rhamnose or what is usually called rhamnose (Rha). ^87^^,^^105^^,^^106^ Entire multiple steps of rhamnose biosynthesis are absent in mammalian cells. To date, neither rhamnose nor the genes responsible for biosynthesis have been found in mammals, especially humans. ^107^^,^^108^ Rhamnose, which is the main component forming the GAC backbone of GAS in the form of poly-rhamnose, whose absence in mammalian cells is a very attractive candidate for the development of a universal GAS vaccine. Considering that the rhamnose biosynthesis pathway is very widespread and conserved in both Gram-positive and Gram-negative bacteria, as well as its absence in mammalian cells, rhamnose or poly-rhamnose-based GAS vaccines will have high protection coverage against various GAS serotypes without causing cross-reactivity in human body tissues.

**Figure 3.**
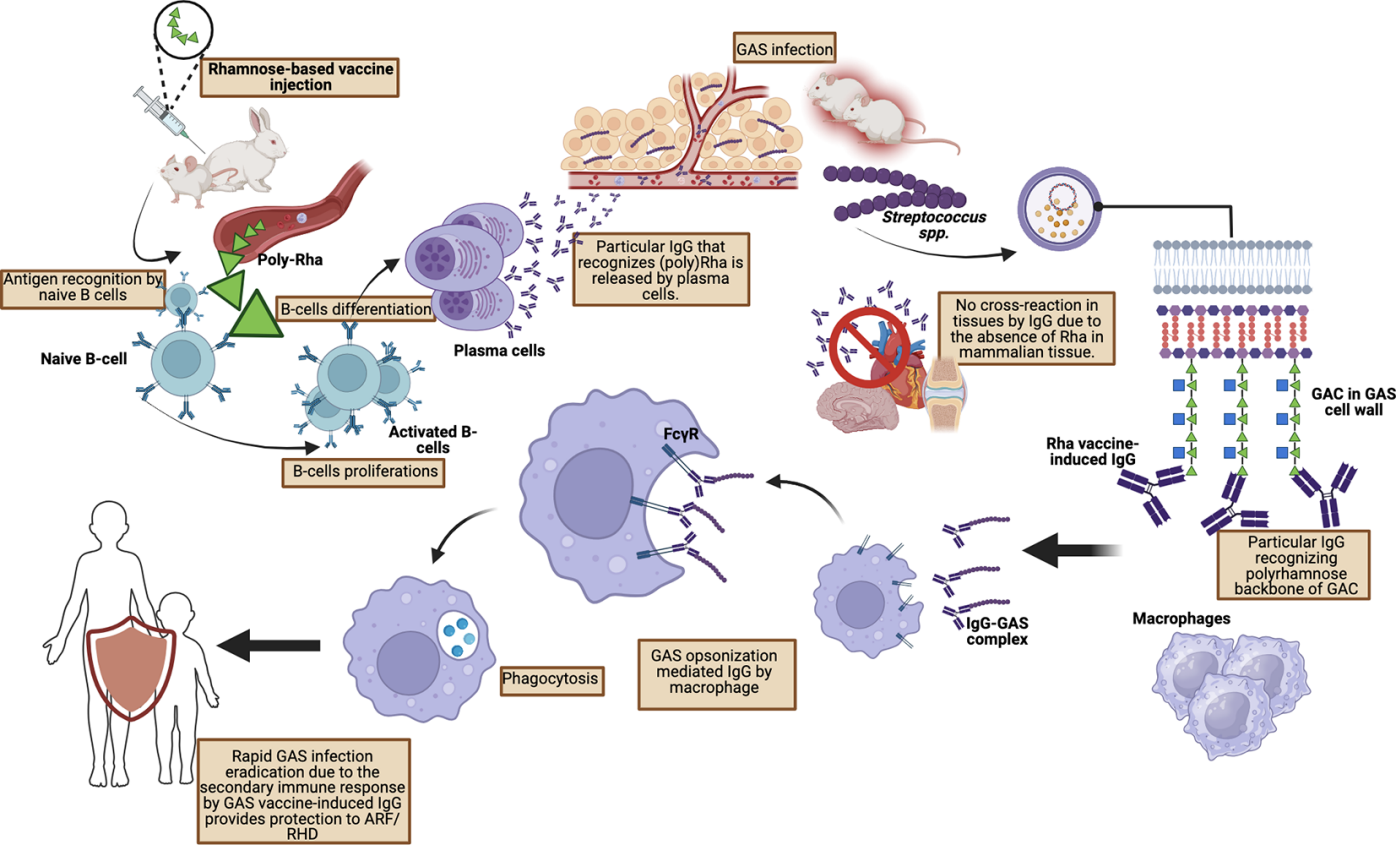
Protective mechanism toward GAS infection by vaccine-mediated IgG by Rhamnose-based vaccine.

A study conducted by van Sorge *et al.* who injected poly-rhamnose (GlcNAc-deficient GAC) showed a significant reduction in binding to human monoclonal antibodies obtained from patients with rheumatic carditis. Furthermore, extensive pre-clinical studies by van Sorge *et al.* using mouse and rabbit models of GAS infection showed that poly-rhamnose backbone injection increased GAC antibodies which promoted opsonophagocytic killing in multiple GAS serotypes ( Figure 3). ^92^

IgG antibodies formed against poly-rhamnose are able to provide passive protection of the murine system against various GAS strains and induce opsonophagocytic activity against the M1 strain as well as eight other strains. ^80^^,^^92^ Recently, a glycoconjugate vaccine has been further developed which grafts synthetic tetra- and hexa-rhamnoside from the GAC backbone onto gold nanoparticles as a vaccine delivery platform. ^109^ Through the vaccine delivery platform, effectiveness and efficacy will increase significantly. Therefore, the rhamnose component is very promising in the development of a safe and effective GAS vaccine. These findings align with an in-vivo investigation performed by Khatun et al., 2021, which included six groups of C57BL/6 mice. According to the immunogenicity study following the third boost, the titer level of antigen-specific IgG was markedly elevated in the intervention control compared to the group that got a phosphate-buffered saline (PBS) injection in the controlled group. Additionally, the safety assessment in this study with 1% dimethyl sulfoxide (DMSO) revealed no adverse reactions, while the application of 10% DMSO in this in-vivo experiment demonstrated it to be non-immunogenic and non-cross reactive with self-tissues. ^95^ An investigation conducted by Michael et. al. in 2018 utilized rhamnan polysaccharide-based glycoconjugates on New Zealand white female rabbits (received 50 μg injection of conjugate with incomplete freuds adjuvant) showing a binding activity of the post-immune serum in immunofluorescence experiments, comparing pre- and post-immune serum (D70) from rabbit RRHV3. Further, the sera from serotype k conjugate-derived animals demonstrated significant killing of homologous k bacteria at elevated titers in comparison to the control sera from protein and admixed controls. The sera from serotype f conjugate-derived mice showed a rise in opsonophagocytic titer, indicating deceased of the homologous serotype strain from pre- to post-immune sera. ^110^ Research conducted by Sowmya *et al.* used recombinant poly-rhamnose backbone (pRha) carried using a carrier in the form of outer membrane vesicles (OMVs) from Escherichia coli bacteria to investigate immunogenicity and efficacy in animal models. The results of this study showed that pRha-OMVs induced specific antibodies that could recognize GAC from *Streptococcus pyogens* and *Streptococcus dysgalactiae* subsp. *Equisimilis.* ^111^ An increase in IgG antibody titer correlates with increased bactericidal killing in the hypervirulent GAS strain M89. Aside from that, the increase in IL-17a in the study’s results indicated that long-term memory immune cells were stimulated after administration of the pRha-OMVs vaccine. ^111^ Furthermore, regarding the safety level of the vaccine, in this study, there was no mention of any cross-reactivity to animal tissue which has been a challenge in the development of GAS vaccines. Over the last few decades, several studies have been conducted to further identify the effectiveness and safety level of rhamnose or poly-rhamnose-based vaccines from GAC ( Table 1).

**Table 1.** Rhamnose (Rha) Based GAS Vaccine from GAC Backbone in Pre-clinical Studies.

Author(s)	Year	Subject	Duration of Treatment	Outcome(s)	Cross-reactivity/Autoimmunity	Reference(s)
Nina M. van Sorge *et al.*	2014	Rabbits and mice	1 week	Stimulate an immunological response that facilitates the opsonophagocytic and immune elimination of several strains of GAS. The resulting antiserum exhibited a remarkably high sensitivity towards the immunizing antigen. The IgG effectively bound to functional wild-type M1 GAS microorganisms, as well as GAS serotypes from 7 other commonly related illness serotypes, enabling neutrophils to eliminate the bacteria. In comparison to an antiserum reactive against wild type, it enhanced the process of neutrophil phagocytosis caused by opsonization and provided immunological passive protection.	None	^92^
Farjana Khatun *et al.*	2021	Pathogen free C57BL/6 mice (female, 4 – 6 weeks old)	42 days	Poly-rhamnose-specific IgG antibodies protected mice from the heterologous M49 GAS serotype and elicited phagocytosis activity induced by opsonization against nine distinctive GAS strains.	None	^95^
Sowmya Ajay Castro *et al.*	2021	Female C57BL/6J mice aged 5 – 6 weeks old.	49 days	Poly-rhamnose (pRha)-containing outer membrane vesicles have been demonstrated to stimulate IgG antibody production. Flow cytometry data revealed that antibody accumulation was significantly higher in blood samples of animals immunized with poly-Rhamnose-OMVs than in control immunized animals. ELISA tests revealed that pRha-OMVs sera recognize Group A Streptococcus strains, including the favored clade M1T1 (a new S. pyogenes emm1 lineage).	None	^111^
Olimpia Pitirollo *et al.*	2023	Female CD1 mice aged 5 weeks old and New Zealand White rabbits Crl:KBL, female	28 days for mice and 35 days for rabbits	The poly-Rha conjugate elicited higher anti-poly-Rha IgG responses in mice and rabbits than the GAC (contained GlcNAc) conjugate.	None	^82^
Nina J. Gao *et al.*	2021	New Zealand White Rabbit and wild-type female CD-1 mice	42 days for mice and 35 days for rabbits	Immunization of New Zealand White rabbits was performed for six of eight strains, the GAS surface binding of antiserum rising towards the GAC-modified vaccine (contained poly-rhamnose only) roughly doubled the amounts of immunoglobulin G binding seen with antiserum raised towards non-modified vaccine. Flow cytometry revealed a 100-400% increase in IgG binding to eight existing wild-type GAS strains of various M protein serotypes (M1, M2, M3, M4, M5, M6, M12, M28). In mice, the triple combination vaccine of GAC-modified vaccine + C5a peptidase + SLO provided remarkable 100% protection towards the lethal challenge. In contrast, injection of GAC modified vaccine alone increases protection by 20%.	None	^80^
Tania Rivera-Hernandez *et al.*	2016	A group of BALB/c rats and A group of transgenic humanized plasminogen rats heterozygous for the human plasminogen gene (AlbPLG1)	28 days	According to ELISA, the response to antigen (GAC lacking GlcNAc) was significantly higher in vaccinated mice than in sham-immunized control mice in both BALB/c mice and transgenic humanized plasminogen mice. Furthermore, vaccinated mice had higher IgG levels for the GAC backbone than control mice. GAS clearance by 60% executing evasive bactericidal assay in mouse skin challenge.	None	^112^
Anna Kabanova *et al.*	2010	A group of CD-1 rats (Female, 5 – 6 weeks old)	35 days	Mice vaccinated and challenged with the M1 serotype had significantly reduced mortality (p-value 0.05) than control mice vaccinated with alum alone, with survival rates ranging from 29% to 50%. IgG antibodies were generated and able to bind to the GAS.	None	^96^

A limitation of this research is that the data presented are derived from pre-clinical trial trials. The effectiveness of the L-Rhamnose-based vaccination utilizing the GAC backbone in enhancing human antibody titers against GAS bacteria remains inconclusive. This pertains to the development of a GAS vaccine originating from a non-M protein derivative, which remains confined to pre-clinical research and has not yet undergone official testing on human beings. Moreover, the scarcity of recent data, particularly research conducted in the past two years on L-rhamnose-based vaccines, has compelled the authors to seek data spanning the last decade or more to present substantial variations in effectiveness and safety regarding the use of L-rhamnose as a vaccine against GAS infection. This paper represents the inaugural effort to exclusively discuss and synthesize diverse research findings regarding the efficacy and safety of L-rhamnose-based vaccines, which may serve as a significantly safer vaccine for Group A Streptococcus (GAS), given the minimal cross-reactivity resulting from the absence of rhamnose components in mammalian tissues, particularly in humans. This paper can advance research to the next stage, specifically enabling the prompt consideration and implementation of clinical trials on humans.

## Conclusion

The Group A Streptococcus (GAS) vaccine based on l-Rhamnose obtained from the poly-rhamnose backbone Group A Carbohydrate (GAC) has shown a protective effect against GAS infection which has been proven through pre-clinical studies which show an increase in IgG antibody titers facilitating and improving opsonophagocytic ability against various GAS strains. In addition, various recent studies have shown that the use of Rhamnose-based vaccines does not show any cross-reactivity in test animal tissue thus the vaccine is able to provide protection against GAS infection without causing ARF attacks due to the absence of cross-reactive antibodies. These findings demonstrate that the l-Rhamnose-based vaccine possesses strong immunogenicity, which effectively protects against GAS infection while maintaining a significantly higher degree of safety. Prevention of GAS infection through effective vaccination will provide protection against ARF attacks and its sequelae, namely RHD, which has become a global burden both in terms of epidemiology and management costs, especially in low- and middle-income countries.

## Data Availability

No data are associated with this article.
