# Supplementary material for: Unveiling the Group A Streptococcus Vaccine-Based L-Rhamnose from Backbone of Group A Carbohydrate: Current Insight Against Acute Rheumatic Fever to Reduce the Global Burden of Rheumatic Heart Disease
Source: F1000Res. 2025 Jan 30;13:132. Originally published 2024 Feb 22. [Version 3] doi: 10.12688/f1000research.144903.3 (PMC11829149; doi:10.12688/f1000research.144903.3)
Supplement: Peer review history

# Reviewer report: Reviewer response for version 3

**DOI:** 10.5256/f1000research.177425.r363558

**Published:** 2025-03-10

**Authors:** Arif Nur Muhammad Ansori

**Affiliations:** 1 Universitas Airlangga, Surabaya, East Java, Indonesia

Approved.

Is the review written in accessible language?

No

Are all factual statements correct and adequately supported by citations?

Yes

Are the conclusions drawn appropriate in the context of the current research literature?

Yes

Is the topic of the review discussed comprehensively in the context of the current literature?

Yes

Reviewer Expertise:

Molecular Biology and Bioinformatics

I confirm that I have read this submission and believe that I have an appropriate level of expertise to confirm that it is of an acceptable scientific standard.

# Reviewer report: Reviewer response for version 3

**DOI:** 10.5256/f1000research.177425.r363559

**Published:** 2025-02-14

**Authors:** Mark Kaddumukasa

**Affiliations:** 1 Makerere University, Kampala, Central Region, Uganda

The revised manuscript and the authors responses addressed the issues that were raised and was given an approval for acceptance. I have no further concerns.

Is the review written in accessible language?

Yes

Are all factual statements correct and adequately supported by citations?

Yes

Are the conclusions drawn appropriate in the context of the current research literature?

Yes

Is the topic of the review discussed comprehensively in the context of the current literature?

Yes

Reviewer Expertise:

NA

I confirm that I have read this submission and believe that I have an appropriate level of expertise to confirm that it is of an acceptable scientific standard.

# Reviewer report: Reviewer response for version 2

**DOI:** 10.5256/f1000research.173666.r343519

**Published:** 2025-01-10

**Authors:** Mark Kaddumukasa

**Affiliations:** 1 Makerere University, Kampala, Central Region, Uganda

The manuscript covers an important topic that requires re-inventing the prevention and control of RHD in LMICs. However, some clarifications are needed to improve the article.

1. Please include a section on limitations pertaining to the techniques and proposed.
2. It would be better to include within the discussion the issue of hypo responsiveness, shielding of protein immune epitopes associated with carbohydrate epitopes and how the best antigens for the proposed vaccines can be selected.  

Is the review written in accessible language?

Yes

Are all factual statements correct and adequately supported by citations?

Yes

Are the conclusions drawn appropriate in the context of the current research literature?

Yes

Is the topic of the review discussed comprehensively in the context of the current literature?

Yes

Reviewer Expertise:

NA

I confirm that I have read this submission and believe that I have an appropriate level of expertise to confirm that it is of an acceptable scientific standard, however I have significant reservations, as outlined above.

# Response

**Published:** 2025-01-22

**Authors:** Ade Meidian Ambari

**Affiliations:** Cardiology and Vascular, Faculty of Medicine, University of Indonesia, Jakarta, Jakarta, Indonesia

The author(s) express gratitude and appreciation for the valuable suggestions and constructive feedback. Consequently, the author(s) will return the amended manuscript addressing the prior review comments and respond to the question.

 1. Please include a section on limitations pertaining to the techniques and proposed.

**Answer:**

 Thank you for the constructive feedback, here we have added the limitation pertaining the techniques to our revised paper.

 "The paper is a comprehensive narrative review; thus, the limitations related to the techniques include a lack of standardized procedures and reproducibility caused by a lack of a defined methodology for searching, selecting, and analyzing literature. Nonetheless, the authors remain to strictly check and scrutinize the articles as sources to ensure high-quality data acquisition."

 2. It would be better to include within the discussion the issue of hypo responsiveness, shielding of protein immune epitopes associated with carbohydrate epitopes and how the best antigens for the proposed vaccines can be selected.  

**Answer:**

 "A further obstacle in advancing polysaccharide-based vaccines and their derivatives is the immunogenicity elicited by polysaccharides. Bacterial polysaccharides typically elicit short-lived T cell-independent responses and largely do not facilitate the development of memory B cells or the generation of long-lived plasma cells. As a result, polysaccharide-based vaccinations and their derivatives typically elicit low-affinity antibody responses and are ineffective in generating long-term, boostable immunological memory. Further, in adults, the administration of polysaccharide vaccines is suboptimal since it fails to elicit immunological memory, avidity maturation, and isotype switching. The majority of antibodies generated are IgM and IgG2, which are ineffective complement activators, therefore diminishing their capacity to defend against infections. As discussed above, a significant issue with polysaccharide vaccines is their inadequate immunogenicity. Based on the limitation, glycoconjugate vaccines were created to enhance the inadequate protection offered by polysaccharide vaccinations. Adjuvants can be categorized into many classes according to their physicochemical features, including protein-based adjuvants (PBAs). An adjuvant is a compound or combination of substances incorporated into a vaccine to augment immunogenicity and elicit an initial innate immune response by stimulating an inflammatory reaction at the injection site. Consequently, an adjuvant amplifies the intensity and longevity of the vaccination's effects while modifying the nature of particular adaptive downstream immune responses to vaccine antigens, without eliciting a specific antigenic response against itself. The covalent attachment of bacterial glycans to carrier proteins in these vaccines enhances the immunogenicity of saccharide antigens by stimulating T cell-dependent B cell responses, resulting in high-affinity antibodies and long-lasting protection. Glycoconjugate antigens may induce a T cell-dependent response, leading to the synthesis of high-affinity antibodies and the formation of carbohydrate-specific memory B cells (MBCs). Consequently, glycoconjugate vaccinations provide protection for young infants (under 2 years of age) and are generally more potent than pure polysaccharide vaccines."

 Further, the content above has been added by the authors to the revised paper in the discussion section.

# Reviewer report: Reviewer response for version 2

**DOI:** 10.5256/f1000research.173666.r335591

**Published:** 2024-10-29

**Authors:** Arif Nur Muhammad Ansori

**Affiliations:** 1 Universitas Airlangga, Surabaya, East Java, Indonesia

This revised article provides a valuable and comprehensive overview of the potential of an L-Rhamnose-based vaccine to prevent Group A Streptococcus (GAS) infections, aiming to reduce acute rheumatic fever (ARF) and the global burden of rheumatic heart disease (RHD). The authors effectively contextualize the issue, emphasizing the challenges of current management strategies, particularly in resource-limited settings, and underline the promise of preventive vaccination approaches.

 The focus on the L-Rhamnose-based vaccine as an alternative to M-protein-based vaccines is scientifically sound, and the article explains well why L-Rhamnose presents a lower risk of triggering cross-reactive antibodies. This perspective is crucial, as it addresses the potential adverse effects associated with M-protein cross-reactivity—a notable concern in previous GAS vaccine developments.

Is the review written in accessible language?

No

Are all factual statements correct and adequately supported by citations?

Yes

Are the conclusions drawn appropriate in the context of the current research literature?

Yes

Is the topic of the review discussed comprehensively in the context of the current literature?

Yes

Reviewer Expertise:

Molecular Biology and Bioinformatics

I confirm that I have read this submission and believe that I have an appropriate level of expertise to confirm that it is of an acceptable scientific standard.

# Reviewer report: Reviewer response for version 1

**DOI:** 10.5256/f1000research.158764.r330060

**Published:** 2024-10-10

**Authors:** Arif Nur Muhammad Ansori

**Affiliations:** 1 Universitas Airlangga, Surabaya, East Java, Indonesia

First of all, this manuscript is good and well-written. However, I may require some comments on the following issues. Basically, this manuscript is acceptable.

 However, in order to improve the readability of the paper by an English editing service as various sentences are mistyped. 

 Title: The title is easy to follow and no mistakes.

 Abstract: This section was well-written and easy to understand. In addition, the study's objective and the state of the art of the study is clear. Furthermore, the keywords should represent the study.

 Discussion: The discussion of the study is lack of information about the important topics. To develop a systematic discussion, the authors should mention the limitation(s) of the study.

 Conclusion: In this part of the paper, the authors have established the conclusion of this study.

 References: Many references are out of guidelines. Please kindly check the references again, do not include the old references, and check the abbreviation of the journals/proceedings. Please mention many high-quality related publications in 2023-2024.

Is the review written in accessible language?

No

Are all factual statements correct and adequately supported by citations?

Yes

Are the conclusions drawn appropriate in the context of the current research literature?

Yes

Is the topic of the review discussed comprehensively in the context of the current literature?

Yes

Reviewer Expertise:

Molecular Biology and Bioinformatics

I confirm that I have read this submission and believe that I have an appropriate level of expertise to confirm that it is of an acceptable scientific standard, however I have significant reservations, as outlined above.

# Response

**Published:** 2024-10-17

**Authors:** Ade Meidian Ambari

**Affiliations:** Cardiology and Vascular, Faculty of Medicine, University of Indonesia, Jakarta, Jakarta, Indonesia

The authors convey their appreciation and gratitude to the reviewers for their constructive assessments and evaluations. The reviewers' suggestion has been optimally implemented, and these enhancements are evident in the latest version of this paper. Furthermore, we have enhanced the quality of grammar and English use by using editing services and utilizing grammar and paraphrasing tools to augment the paper's readability.

 Title: The title is easy to follow and no mistakes.

 Response: Thank you for the provided assessment.

 Abstract: This section was well-written and easy to understand. In addition, the study's objective and the state of the art of the study is clear. Furthermore, the keywords should represent the study.

 Response: Thank you for your insightful evaluation and feedback. The author modified the keywords to more accurately reflect the study.

 Discussion: The discussion of the study is lack of information about the important topics. To develop a systematic discussion, the authors should mention the limitation(s) of the study.

 Response: Thank you for the feedback. The authors enhance the quality of the Discussion section by including and expanding on different prior research findings, particularly those concerning the efficacy and safety of the Rhamnose-based GAS Vaccine, which is key to this study topic. In addition, the authors have included the study's limitations in the final paragraph of the discussion section. The authors faced challenges in incorporating relevant research data published in the past two years, which pertains to the study's shortcomings. The search was extended to encompass a minimum of the past decade.

 Conclusion: In this part of the paper, the authors have established the conclusion of this study.

 Response: Thank you for the provided assessment.

 References: Many references are out of guidelines. Please kindly check the references again, do not include the old references, and check the abbreviation of the journals/proceedings. Please mention many high-quality related publications in 2023-2024.

 Response: I appreciate your constructive criticism. The authors have assessed and amended all references cited in the manuscript according to metadata from the reference management software (Mendeley). Furthermore, we have eliminated several outdated references deemed non-essential (material remains accessible in other publications) (e.g., Giraud MF, Naismith JH. The rhamnose pathway. Curr Opin Struct Biol. 2000 Dec;10(6):687–96.), and the authors have incorporated recent studies published in 2023.
